# Displacement of the third molar bud to the sublingual space during open reduction and internal fixation of mandibular angle fracture: A case report

**DOI:** 10.1002/ccr3.3451

**Published:** 2020-10-30

**Authors:** Reza Sharifi, Lotfollah Kamaliehakim, Narges Matloubi

**Affiliations:** ^1^ Department of Oral and Maxillofacial Surgery Cranio Maxillofacial Research Center Shariati Hospital Tehran university of Medical Sciences Tehran Iran; ^2^ Tehran university of Medical Sciences Tehran Iran

**Keywords:** impacted tooth, mandibular fracture, third molar, tooth displacement

## Abstract

This case could remind surgeons to consider the possibility of displacement of the impacted third molar in the fractured line to adjacent spaces, during open reduction and internal fixation of mandibular angle fracture.

## Graphical abstract

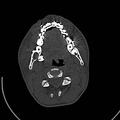

## 1 INTRODUCTION

This case could remind surgeons to consider the possibility of displacement of the impacted third molar in the fractured line to adjacent spaces, during open reduction and internal fixation of mandibular angle fracture. Furthermore, this can justify the need to remove the third molar in the fracture line.

The fracture of the mandibular angle is the most common mandibular fracture in most statistical studies. The presence of an impacted mandibular third molar is one of the reasons for the weakening of the mandible in this area which increases the risk of angle fractures.^1^ The presence of teeth in the fracture line can disrupt the reduction of bone fragments. Nonetheless, there is no need to extract the tooth at the absence of disruption.^2^

According to R. Seemann study, the complications of mandibular angle fractures include infection, delayed recovery, improper recovery, neurosensory disorders, and inappropriate occlusion.^3^ To the best of our knowledge, the displacement of the impacted mandibular third molar to adjacent spaces after ORIF has not been reported in any study.

In the present study, we have introduced the very rare complication of the treatment of mandibular angle fractures and its management.

## 2 CASE REPORT

Our case was a 16‐year‐old patient who had suffered mandibular trauma due to combat sport. He was referred to the Teaching Shariati Hospital affiliated with Tehran University of Medical Sciences.

The patient had no systemic disease or allergies and complained of pain and jaw dislocation. In clinical examination, the swelling was detected in the left side of mandible, and asymmetry was evident on the left side of the face due to mandibular division. The bone step was touched on the right angle. Crossbite and open bite from teeth 2 to 7 on the left and anterior open bite and mandibular shift to the left side were observed. There were no neurosensory disturbances.

**Figure 1:**
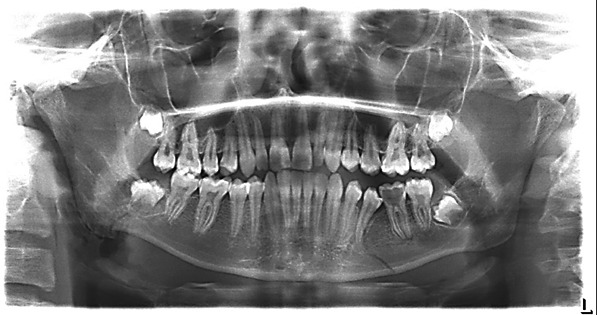
Panoramic view of fractured areas. The third molar bud in the fractured angle without displacement

After clinical examination and preparing OPG and PA Cephalometric radiographs, fractured area of the right angle and mandibular body fracture on the left side with slight displacement was detected (Figure 1). The third molar bud was vertically present in the fractured area without displacement.

The patient underwent open reduction and internal fixation (ORIF) surgery by fastening Eric's arch bar and achieving proper occlusion in the IMF. Two mini plates in the body area and one 4‐hole mini‐plate on the external oblique ridge for the fixation of mandibular angle based on Champy treatment protocol were inserted. During the operation, a suitable key and keyhole existed for anatomical reduction, and the third molar bud was not exposed; In addition, it was sought to prevent the destruction of the fracture edges that were properly reduced; accordingly, no attempt was made to remove the third molar tooth.

**Figure 2:**
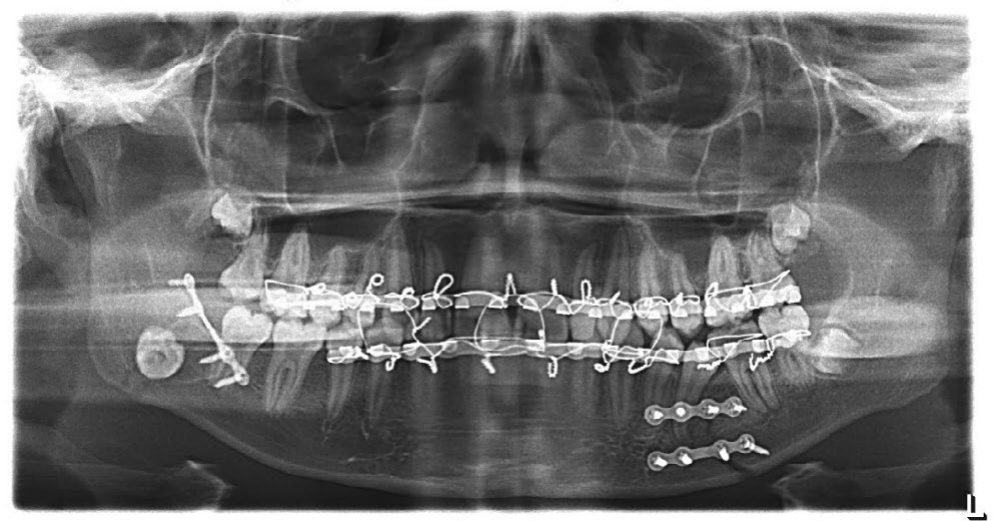
Postoperation radiographs, the displacement of the third molar bud in the panoramic view

**Figure 3:**
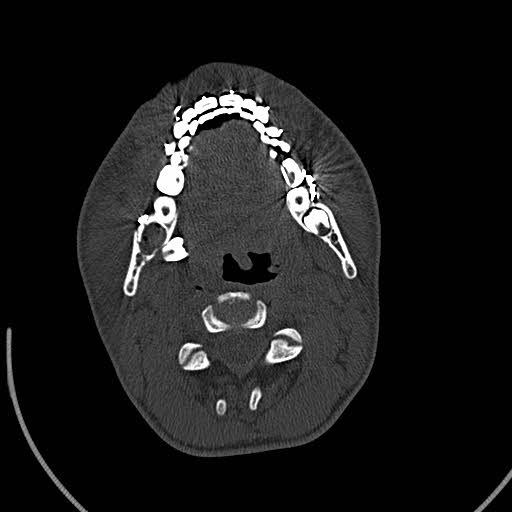
Computed tomography (CT) scan axial view. The third molar bud was displaced to the sublingual space

**Figure 4:**
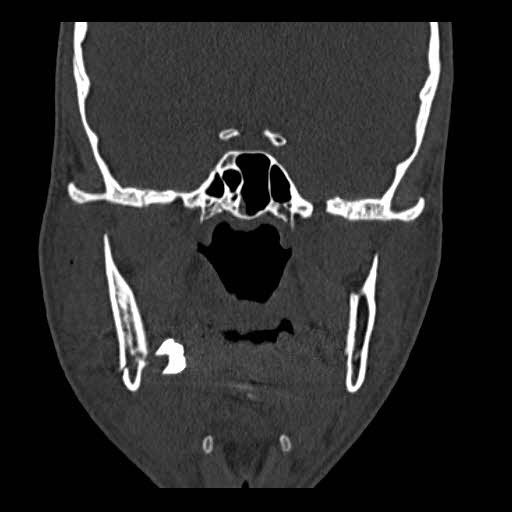
Computed tomography (CT) scan coronal view. The third molar bud was displaced to the sublingual space

After preparing the postoperation radiographs, the displacement of the third molar bud was observed in the panoramic view (Figure 2). A computed tomography (CT) scan was performed to examine the position of the third molar (Figures 3 and 4). According to the CT scan, the third molar bud was displaced to the sublingual space due to a fracture of the lingual cortex. Due to the possibility of infection, threat to the repair of the fractured area and foreign body reaction, it was decided to remove the displaced tooth immediately.

The patient was taken back to the operating room two days later, and the displaced tooth was found and removed under anesthesia with oral access from the previous incision area, by opening two distal screws of Champy plate and briefly opening and exploring the area of the mandibular angle. The appropriate reduction was obtained and fixation was performed in the same points as before. During the follow‐up, no abnormalities were observed in neurosensory status and occlusion after 1 week.

## 3 DISCUSSION

Tooth displacement to adjacent spaces which has been reported by many authors is one of the rare complications of mandibular third molar surgery.^4^ Due to a fracture of the thin lingual cortex, a fragmentation of the root or entire tooth may be displaced to the sublingual, submandibular, and pterygomandibular, and more rarely to the pharyngeal lateral space.^5^

This report presented the case of displacement of the third molar tooth to the sublingual space during the reduction of angle fracture.

Mandibular angle fractures account for about 30% of single mandibular fractures, and concomitant body and angle or parasymphysis and angle fractures are very common.^6^ The high prevalence of angle fractures can be justified on the ground of the weakening of the bone in this area due to the presence of impacted third molar, a thinner cross‐sectional area, and the biomechanical position can be considered the lever area.^7^

The angle fracture line usually affects the third molar. According to research conducted by WOLUJEWICZ et al, the third molar can help maintain fracture reduction if it has a horizontal and mesioangular position. Vertical teeth, on the other hand, may interfere with reduction and further displacement of fragmentations; consequently, it is recommended to remove the vertical teeth before reduction.^2^ According to a study performed by Ellis, the third molar tooth must be removed during the operation if it is infectious, decayed, fractured, 4‐degree mobility, or at the presence of an apical lesion, otherwise, the surgeon will decide to remove it.^8^ In the present study, the third molar bud was completely impacted and due to the lack of disturbance in the reduction during the operation, no attempt was made to remove it.

The thickness of the lingual cortex in the third molar area is very thin resulting in its fracture and the displacement of the tooth to adjacent spaces.^9^ In this study, the patient underwent standard radiographs before surgery to check for a mandibular fracture, and the third molar was in its proper position in panoramic view. The textbooks did not refer to the possibility of the displacement of the impacted third molar to the adjacent spaces during the reduction, then the surgeon did not expected this complication.

The third molar displaced in the surrounding space has the potential for infection and foreign body reaction. In normal cases, the removal can be performed immediately or after 3‐4 weeks and the fibrosis around the tooth and its immobilization in the area, depending on the surgeon's opinion.^10^

In this case, due to the possibility of disruption in the recovery of the angle fracture area and the occurrence of more complications in the absence of removal, the decision was made to remove the displaced tooth immediately. Due to the fact that the tooth was displaced to the sublingual space, it was removed via the oral approach.^11^

Due to the proximity of the lingual nerve, assiduous attention should be devoted to maintain and not damage the nerve during exploring. In this patient, with undivided attention, no neurosensory damage was observed in the patient.

## 4 CONCLUSION

The experience obtained from this case reminds oral and maxillofacial surgeons to consider the possibility of displacement of the impacted third molar in the fractured line to adjacent spaces, during open reduction and internal fixation of mandibular angle fracture. Furthermore, this can justify the need to remove the third molar in the fracture line.

## CONFLICT OF INTEREST

None declared.

## AUTHOR CONTRIBUTIONS

Reza Sharifi: Carried out the experiment. Lotfollah Kamaliehakim: contributed to sample preparation. Narges Matloubi: wrote the manuscript in consultation with other authors.

## ETHICS STATEMENT/CONFIRMATION OF PATIENT'S PERMISSION

This clinical case report was approved by the ethical committee of Shariati Hospital as there is no identifying information in the report. The patient gave his informed consent.

## References

[1] Giovacchini F , Paradiso D , Bensi C , Belli S , Lomurno G , Tullio A . Association between third molar and mandibular angle fracture: A systematic review and meta‐analysis. J Cranio‐Maxillofac Surg. 2018;46(4):558‐565. 10.1016/j.jcms.2017.12.011 29459187

[2] Wolujewicz MA . Fractures of the mandible involving the impacted third molar tooth: an analysis of 47 cases. Br J Oral Surg. 1980;18(2):125‐131. 6934803 10.1016/0007-117x(80)90028-1

[3] Seemann R , Schicho K , Wutzl A , et al. Complication rates in the operative treatment of mandibular angle fractures: a 10‐year retrospective. J Oral Maxillofac Surg. 2010;68(3):647‐650. 20171484 10.1016/j.joms.2009.07.109

[4] Khalil HAM , Hasanen AM . Iatrogenic fracture of lingual plate of bone and Displacement of Mandibular Third Molar into Submandibular Tissue Space. A Case Report. Egyptian Dental Journal. 2020;66 (2):837–840. 10.21608/edj.2020.26054.1075

[5] Huang C , Zhou C , Xu M , Zou D . Risk factors for lingual plate fracture during mandibular third molar extraction. Clin Oral Investig. 2020;24(11):4133–4142. 10.1007/s00784-020-03286-5 32356209

[6] Rashid A , Eyeson J , Haider D , van Gijn D , Fan K . Incidence and patterns of mandibular fractures during a 5‐year period in a London teaching hospital. Br J Oral Maxillofac Surg. 2013;51(8):794‐798. 23735734 10.1016/j.bjoms.2013.04.007

[7] Ellis IE . Treatment methods for fractures of the mandibular angle. Int J Oral Maxillofac Surg. 1999;28(4):243‐252. 10416889

[8] Ellis E III . Outcomes of patients with teeth in the line of mandibular angle fractures treated with stable internal fixation. J Oral Maxillofac Surg. 2002;60(8):863‐865. 12149727 10.1053/joms.2002.33852

[9] Huang IY , Wu CW , Worthington P . The displaced lower third molar: a literature review and suggestions for management. J Oral Maxillofac Surg. 2007;65(6):1186‐1190. 17517304 10.1016/j.joms.2006.11.031

[10] Bozkurt P , Erdem E . Management of upper and lower molars that are displaced into the neighbouring spaces. Br J Oral Maxillofac Surg. 2017;55(9):e49‐e52. 28735634 10.1016/j.bjoms.2017.07.001

[11] Di Nardo D , Mazzucchi G , Lollobrigida M , et al. Immediate or delayed retrieval of the displaced third molar: a review. J Clin Exp Dentistr. 2019;11(1):e55. 10.4317/jced.55379 PMC6343984 30697395

